# A prefix and attention map discrimination fusion guided attention for biomedical named entity recognition

**DOI:** 10.1186/s12859-023-05172-9

**Published:** 2023-02-08

**Authors:** Zhengyi Guan, Xiaobing Zhou

**Affiliations:** School of Information Science and Engineering, Yunnan University, Kunming, China

**Keywords:** Biomedical named entity recognition, E-BioBERT, PAMDFGA, Word-pair relation classification

## Abstract

**Background:**

The biomedical literature is growing rapidly, and it is increasingly important to extract meaningful information from the vast amount of literature. Biomedical named entity recognition (BioNER) is one of the key and fundamental tasks in biomedical text mining. It also acts as a primitive step for many downstream applications such as relation extraction and knowledge base completion. Therefore, the accurate identification of entities in biomedical literature has certain research value. However, this task is challenging due to the insufficiency of sequence labeling and the lack of large-scale labeled training data and domain knowledge.

**Results:**

In this paper, we use a novel word-pair classification method, design a simple attention mechanism and propose a novel architecture to solve the research difficulties of BioNER more efficiently without leveraging any external knowledge. Specifically, we break down the limitations of sequence labeling-based approaches by predicting the relationship between word pairs. Based on this, we enhance the pre-trained model BioBERT, through the proposed prefix and attention map dscrimination fusion guided attention and propose the E-BioBERT. Our proposed attention differentiates the distribution of different heads in different layers in the BioBERT, which enriches the diversity of self-attention. Our model is superior to state-of-the-art compared models on five available datasets: BC4CHEMD, BC2GM, BC5CDR-Disease, BC5CDR-Chem, and NCBI-Disease, achieving F1-score of 92.55%, 85.45%, 87.53%, 94.16% and 90.55%, respectively.

**Conclusion:**

Compared with many previous various models, our method does not require additional training datasets, external knowledge, and complex training process. The experimental results on five BioNER benchmark datasets demonstrate that our model is better at mining semantic information, alleviating the problem of label inconsistency, and has higher entity recognition ability. More importantly, we analyze and demonstrate the effectiveness of our proposed attention.

## Background

As the number of biomedical articles and resources increases, searching and extracting valuable information becomes challenging. Researchers consider a variety of information sources to transform unstructured textual data into refined knowledge to improve research efficiency. Manual annotation and feature generation by biomedical experts are inefficient because they involve complex processes [1]. Therefore, deep learning (DL) and natural language processing (NLP) are particularly important for biomedical text mining and computational data analysis. Valuable information such as relationships between objects requires us to identify meaningful terms from the text. A meaningful term or phrase in a domain that can be distinguished from similar objects is called a named entity (NE) [2]. Named entity recognition (NER) [3] has become a mature technology in mining medical text terms because it is one fundamental task for natural language processing, which aims to recognize named entities (NEs), such as person, location, disease from raw text and classify them into pre-defined categories [4]. Over the past few decades, NER has attracted a great deal of attention owing to its importance in downstream tasks such as entity linking [5], question answering [6], and relationship extraction [7]. In the biomedical field, biomedical named entity recognition (BioNER) also acts as a fundamental task in biomedical text mining that aims to automatically recognize and classify biomedical entities (e.g. genes, proteins, chemicals and diseases) from biomedical text. Although medical NER is a fundamental upstream task, many difficulties remain. That’s because most of the medical literature is disorganized. Medical texts contain some special features, such as the publication of a large volume of medically relevant disease terminology (such as “adenomatous polyposis coli ”), the publication of some chemicals in letters and numbers (such as “CD-832 ”), a number of medical professional abbreviations (such as “SYN”), and BioNEs constantly increase with new discoveries (e.g. COVID-19 is new.). The specificity of medical texts increases the difficulty of treating NER as a sequence labeling problem. Besides, unlike those public domain named entity recognition tasks, BioNER is more challenging due to the naming complexity [8], lack of large-scale labeled training data [9], domain knowledge [8, 10], data privacy [11], and some ethical concerns [12]. These various factors bring limitations and challenges to solving BioNER. With the development of machine learning, some researchers have traditionally used a variety of natural language processing tools and domain knowledge to solve BioNER problems through well-designed feature engineering [13–15]. Since feature engineering relies on models and domain-specific knowledge, there has been a lot of research on BioNER over the past few decades, ranging from traditional feature-based approaches to recent deep learning-based neural approaches.

In recent years, BioNER methods based on DL and NLP have attracted more and more attention due to their excellent performance because deep learning-based approaches typically do not require manually labeling features. It automatically learns useful features from the sentences. Furthermore, advances in deep learning techniques used in NLP have enabled advances in biomedical text mining models. In the NLP field, a deep learning-based approach transforms text into embeddings and then extracts useful features from these embeddings for biomedical entity recognition. So choosing a suitable feature encoder has always been the most important step in NLP. From 2017 to 2022, the research on BioNER is roughly divided into the following several categories, methods based on various neural networks [16–18], pre-trained models [19, 20], external knowledge [10, 21], and multi-task learning [22–26]. For example, some studies use neural network models to generate high-quality features have become prevalent in solving BioNER tasks [16]. The feature extractors of neural networks are usually convolutional neural network [27] (CNN), long short term memory networks [28] (LSTM), bi-directional LSTM [29] (BiLSTM), or a combination of various neural networks. Machine learning-based conditional random field [30] (CRF) is often used as a classifier in conjunction with these feature extractors. Considering the correlation between neighboring labels, CRF can obtain the global optimal label chain for a given sequence. For instance, BiLSTM-CRF [16] is the most common architecture for BioNER using the deep learning method [31]. Since 2018, large-scale pre-trained language models (PLMs) are proved to be effective in many NLP tasks. Integrating or fine-tuning pre-trained language model embeddings for BioNER is becoming a new paradigm. Pre-trained models like BERT [19] show the effectiveness of the paradigm of first pre-trained an language model on the unlabeled text then fine-tuning the model on the down-stream NLP tasks [32]. Therefore, Lee et al. [20] proposed a variant of BERT, namely, BioBERT [20], for the biomedical domain, which is pre-trained on large raw biomedical corpora and achieves state-of-the-art performance in BioNER [10]. It was hard to beat the performance of BioBERT until recently when someone tries to use external knowledge and multi-task learning to improve the performance of BioNER [10, 21–26]. The recent SoTA model on BioNER in some datasets using multi-task methods are proposed by Tong et al. [26] and Chai et al. [25]. Tong et al. [26] try to combine BioBERT and multi-task by designing three auxiliary classification tasks and one main BioNER task to explore multi-granularity information in the dataset. The multiple loss functions of multiple tasks are jointly trained by assigning different fixed weight coefficients. Their multi-task model is hard parameter sharing. Different from them, Chai et al. [25] select 14 datasets containing 4 types of entities for training and evaluate the model on specific task, which realizes the multi-level information fusion between the underlying entity features and the upper data features. Different from the above models, Tian et al. [10] utilize additional syntactic knowledge to enhance BioNER for the first time. However, these methods have major disadvantages. For example, although multi-task learning is an effective approach to guide the language model to learn task-specific knowledge [33], the relationships between different BioNER tasks are often difficult to consider comprehensively due to differences among different datasets. Besides, multi-task learning is not conducive to model training because loss between different tasks may conflict, resulting in mutual consumption cancellation, or even negative transfer phenomenon, which makes us hard to balance the joint training process of all tasks. As for the methods leveraging additional knowledge, the disadvantages are also obvious: (1) acquiring external knowledge is labor-intensive (e.g., knowledge base) [34, 35] or computationally costly (e.g., dependency); (2) Integrating external knowledge adversely affects end-to-end learning and compromises the generality of DL-based systems [31]. Although some external syntactic information is easier to obtain through off-the-shelf NLP toolkits like spaCy or Stanford CoreNLP Toolkits [10, 17, 36], the text structure of BioNER is usually complex, and it is difficult to integrate general syntactic structure information into multiple BioNER datasets. Finally, the above methods are all based on sequence labeling which means the label of each word is predicted independently based on a context-dependent representation, regardless of its neighbors. We believe that ignoring the neighbors around entity words in sequence labeling will weaken the recognition ability of special medical words. And the complexity of biomedical terminology brings challenges and difficulties to sequence labeling. Different from all the above methods, we use a new prediction mode, namely word-pair relation classification, instead of the sequence tagging-based mode. We will introduce the differences and advantages between our novel approach and sequence tagging in Related work and Method sections. And we enhance the pre-trained BioBERT model with the proposed attention in a way that does not require additional knowledge to improve the performance of recognizing complex medical terms. To summarize, this paper makes the following contributions:

- We first use a word-pair relation classification to solve the difficulty of sequence labeling for BioNER tasks.
- We design an attention mechanism guided by fused prefix and attention map discrimination to enhance the BioBERT. Our proposed attention can be easily integrated to Transformer-based PLMs [37], which allows initialization from PLMs without introducing any new parameters, and only affects fine-tuning of standard model parameters.
- We evaluate the proposed model on five BioNER datasets to demonstrate its generality.

## Related work

Sequence labeling (also known as sequence tagging) is to input a string and output the sequence corresponding to each character in the string. Complete word segmentation through sequence tagging, that is, mark a character, whether it is the beginning, end, or middle part of a word. Sequence labeling has long been used to model and solve NLP tasks [38], including the BioNER tasks. Sequence labeling is a relatively simple NLP task, but it can also be the most basic task since it covers a wide range of characters, which can solve a series of problems in character classification, such as word segmentation, part-of-speech tagging, named entity recognition, relation extraction and so on. In this method, we need training a sequence labeling model by assigning and designing a label with some tagging schemes for each token in a given sequence. However, sequence labeling methods have many disadvantages in solving the BioNER tasks. First, designing a general labeling scheme for all BioNER subtasks is difficult and labor-intensive [39]. Data annotation plays a crucial role in establishing benchmarks and ensuring that the correct information is used to learn BioNER models. Getting accurate labels requires not only time but also expertise. However, labelling errors are almost unavoidable, and wrong labels can lead to label inconsistencies between subsets of the labeled data (e.g., training and test sets, or multiple training subsets) [40]. Different tagging schemes such as BIO (i.e., B-begin, I-inside, O-outside) or BIOES (i.e., B-begin, I-inside, O-outside, E-end, S-single) usually have a great impact on the performance of the model. Second, for sequence labeling methods, it is difficult to capture the relationship between entity tokens because labels are usually independent and unrelated. Therefore, modeling between entity words has always been the bottleneck of BioNER. For example, “CD-832” is an important and indivisible terminology of chemical entity. However, in the model based on sequence tagging, even if BioBERT model is used, it may only recognize several entity words in these three words, and even recognize three words in the form of “BIB” (In other words, the label consistency problem still remains.). As a result, the model finally mistakenly divides this complete chemical entity word into two biomedical concepts, namely “CD-” and “832”. This is incorrect and unfriendly to biomedical literature mining. At last, entity sparsity problem often exists in the BioNER because sentences in BioNER datasets are too long but contain few entity words. Sequence tagging-based models such as BioBERT+Softmax often cause the classifier to classify entities into too many pre-defined categories, which will lower the probability accuracy of the model. And traditional sequence labeling methods require a specific-label output layer based on PLMs, which is not conducive to generalization [41]. Sequence labeling methods can not better solve BioNER tasks, which provides research interest and value for our efforts to use a more efficient prediction pattern to solve BioNER related tasks.

Besides, in the last 3 years, Transformer-based [37] pre-trained models such as BERT [19] is designed to pre-train language representations on large-scale unlabeled datasets, which has achieved remarkable success on many biomedical text mining tasks [42]. Unlike traditional word embeddings such as Word2Vec [43] and GloVe [44], pre-trained models can capture the meaning of words in different contexts. As we all know, a pre-trained language model like BERT is normally a large-scale and powerful neural network trained with huge amounts of data samples and computing resources [45, 46]. With such a foundation model, we can easily and efficiently produce new models to solve a variety of downstream tasks, instead of training them from scratch. PLMs’ key feature is the self-attention mechanism. Relying on this attention mechanism, Transfomer can contextualize the input and provides an alternative to conventionally used recurrent neural networks (RNN) [47]. However, although Transformer has performed remarkably well, standing on the multi-headed dot-product attention which fully takes into account the global contextualized information, there are still many problems with this attention mechanism [48]. For example, people find that these language models exhibit simple attention patterns [49–51]. For example, Kovaleva et al. [47] find that 40% of heads in a pre-trained BERT model simply pay attention to the delimiters, such as ‘[CLS]’ and/or ‘[SEP]’. This means that different heads of different self-attention layers of BERT always exhibit limited and redundant attention patterns [51–53]. Michel et al. [52] find that a large percentage of attention heads can be removed at test time without significantly impacting performance. Experiments of Raganato et al. [54] conclude that many patterns learned from the encoder’s attention head just reflect contextual location information. Some studies have also found that skimming irrelevant parts or tokens in the input sequence and keeping the hidden state unchanged can speed up the inference of the Transformer will remove the partial redundancy of the Transformer [42, 55]. To deal with these problems, many studies are devoted to changing the distribution of attention heads by the following three research strategies. The first research strategy focuses on the interpretation of networks, namely analyzing attention mechanisms and interpretability of weights and connections [49, 54, 56] or try to to guide the attention mechanism by external information [57, 58]. The second research strategy is that some researchers have explored how to improve the inference efficiency of Transformer by pruning attention heads during inference due to excessive redundancy of the information learned among multiple heads of the Transformer, resulting in excessive network parameters [52–54, 59, 60]. The last research strategy is some researchers make the Transformer focus on the local area by modifying the attention formula [61, 62] and explore the opportunity on the dynamic reduction of input sequence length to reduce time complexity of Transformer [63–66]. Our attention in this paper belongs to the first research line, which is an guided attention but we don’t need additional information. Although the Transformer’s self-attention head learns partially redundant information and unimportant patterns, this will vary from dataset to dataset. It is difficult for us to judge which information learned by the head is useful for different datasets. We want to guide self-attention learning in the global attention map without modifying the self-attention computation and introducing external information. Meanwhile, notably, although the paradigm of pre-training and fine-tuning has achieved remarkable results on many NLP tasks, this paradigm can not better stimulate the knowledgeability of the pre-trained models. Some recent efforts on probing knowledge of PLMs show that, by writing some natural language prompts (also known as templates), we can induce PLMs to complete factual knowledge [67, 68]. For example, GPT-3 [69] further utilizes the information provided by prompts to conduct few-shot learning and achieves awesome results. Then, prompt learning has been introduced [68]. The operation of the prompt is different from the previous fine-tuning based on the PLMs paradigm. In prompt learning, especially for text classification, downstream tasks are formalized as equivalent cloze-style tasks, and PLMs are asked to handle these cloze-style tasks instead of original downstream tasks. For example, compared with conventional fine-tuning methods, prompt learning needs to reconstruct the input data through the template, so that the predicted content is also embedded in the input data, and the mask language model-like [19] (MLM) method can be used to learn the label information. Prompt learning has two types of prompts, namely discrete prompt and continuous prompt (also known as prefix). Prompt learning has been proven to have good results on some simple NLP tasks, including text classification, natural language inference, and so on. But unfortunately, prompt learning may perform poorly compared to fine-tuning on several hard sequence tasks such as information extraction and sequence tagging [70]. Because the template-based prompt method needs to iterate over all spans, the complexity is very high [41]. Later, Liu and Li et al. [69, 71, 72] proposed prompt tuning, an idea of tuning only the continuous prompts. They try to apply continuous prefix for every layer of the pre-trained model [70]. In other words, prefix tuning prepends a sequence of continuous task-specific vectors to the input [71]. This is also a great inspiration for our work.

In this work, instead of treating the BioNER task as a sequence labeling problem, we formulate it as a word pairs relation classification problem [39]. To the best of our knowledge, there is currently no specific research for BioNER by using this research mode. Furthermore, we are the first to explore enhancing PLMs based on this new research mode in BioNER. And we believe that generating continuous prompts can provide certain guided semantic information for word-pair representation on the BioNER datasets because word-pair relationship classification can be seen as a dimensionality reduction operation for sequence labeling. We are committed to designing a more diverse attention mechanism based on prompt tuning, which can make the representation of the same head as similar as possible while the distribution of different heads is as diverse as possible. In this way, the probability of entity words being noticed will increase. This kind of attention can enrich the diversity of multi-head attention at different layers of PLMs without introducing external knowledge, syntax trees and modifying the self-attention. We design this attention as a unified auxiliary task, which can be applied to any efficient model (This is our future work.). Therefore, we propose the prefix and attention map discrimination fusion guided attention (PAMDFGA). As far as we know, in the BioNER filed, no researchers have done similar prompt and attention guiding research. Our work is the first to guide the distribution of pre-trained models by using the prompt to solve the BioNER problem. The following section will introduce how PAMDFGA guides our model in detail.

## Method

### Task definition

**Fig. 1:**
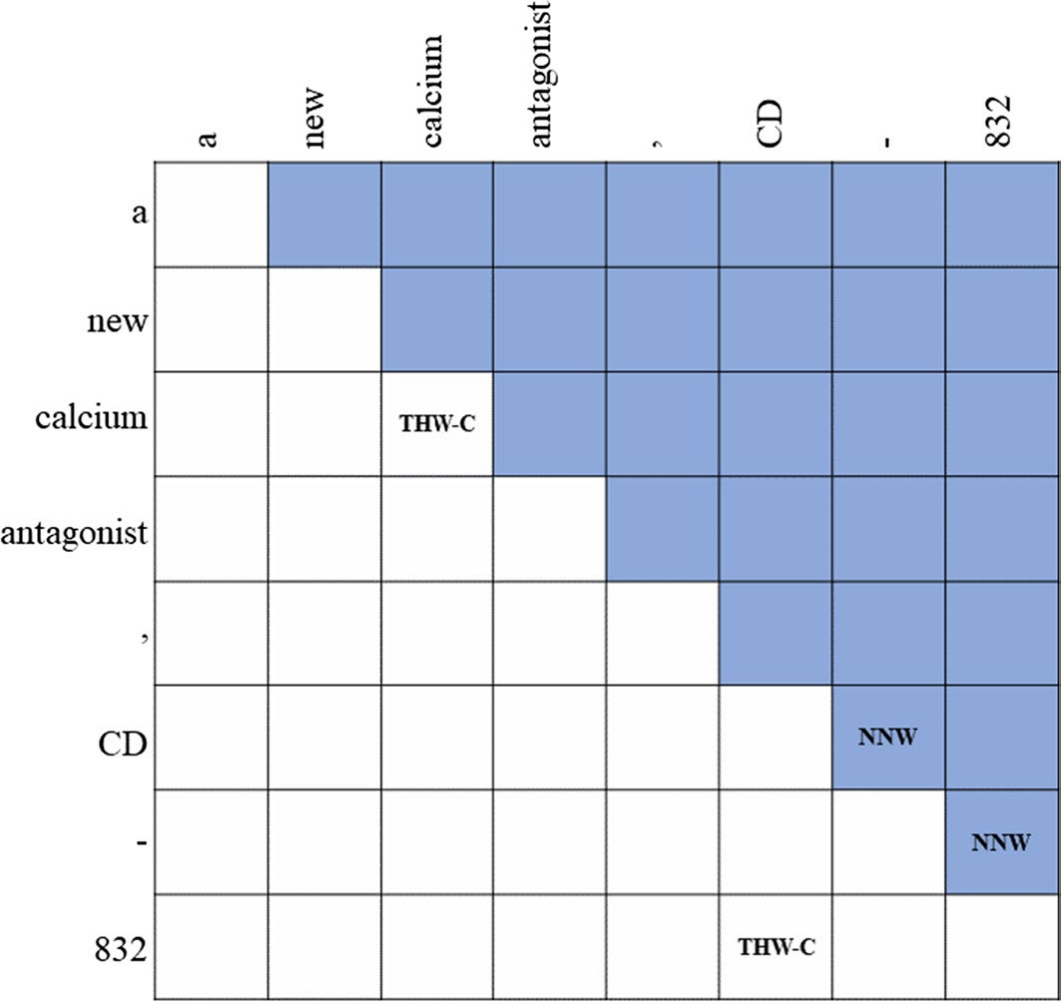
An example to show our relation classification method for BioNER. NNW denotes the Next-Neighboring-Word relation and THW-C denotes the Tail-Head-Word relation that exists in a “Chemical” entity

Formally, for a sequence labeling task, given a sequence of tokens $s =$ $\langle$ $s_{1}$, $s_{2}$, ... , $s_{n}$ $\rangle$, PLMs are to output a list of tuples $\langle$ ${l_s}$, ${l_e}$, *t* $\rangle$. Here, ${l_s}$ $\in$ [1, N] and ${l_e}$ $\in$ [1, N] are the start and the end indexes of a named entity mention. *t* is the entity type from a pre-defined category set [31]. However, in our model, we do not use this prediction mode. This method cannot better mine the entity information of biomedical text, so we explore a model that can strengthen the attention of biomedical entities. Inspired by Li et al [39], our task is to predict the relationship between biomedical word pairs. Specifically, we design two pointer-like word-pair representations for BioNER datasets, namely Next-Neighboring-Word (NNW) and Tail-Head-Word (THW) for BioNER. The NNW relation addresses entity word identification, indicating if two argument words are adjacent in an entity, while the THW relation accounts for entity boundary and type detection, revealing if two argument words are the tail and head boundaries respectively. We give an example as demonstrated in Fig. 1 for a better understanding. Our task aims to extract the relations $\mathfrak {R}$, between each word pairs (${x_i}, {x_j}$), where $\mathfrak {R}$ is pre-defined, including None, NNW, and THW-$\star$ (“$\star$” represents the type of the entity.). As shown in Fig. 1, “CD-832” is a complete entity of chemical. This whole entity includes two relations NNW (CD$\rightarrow$-, and -$\rightarrow$832 ) and THW-C (832$\rightarrow$CD). If there is no relationship between word pairs, we set it to None. Therefore, a 2-dimensional grid for word pairs is constructed in Fig. 1. If an entity such as “calcium” has only one word we set it to THW-C. To avoid the sparsity of relation instances, NNW and THW-C relations are tagged in the upper and lower triangular regions. Our model needs to predict the relation between all word pairs and finally decode it. Through this method, we can better capture the semantic relationship between adjacent entities. With this constructed grid, we don’t have to design a label for each word [39].

### Model

**Fig. 2:**
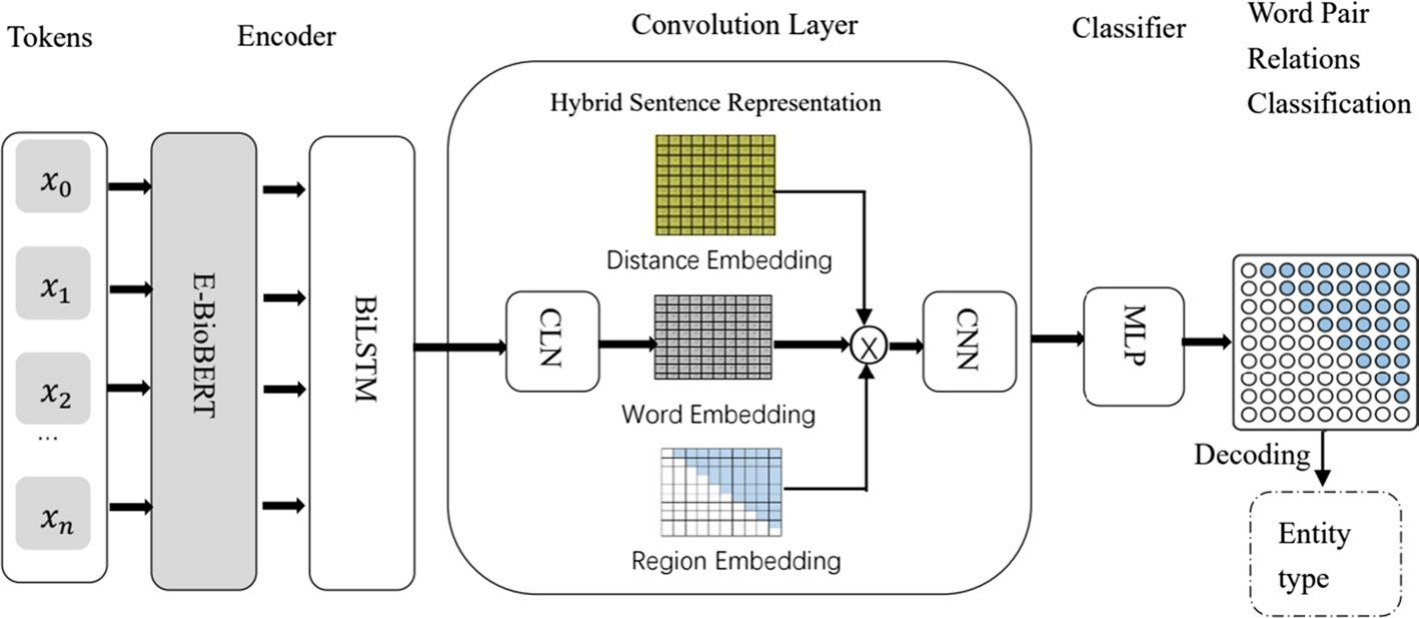
Our model

In this section, we will present the overall model architecture proposed in our method. The architecture of our framework is illustrated in Fig. 2. It mainly consists of three components. First, the enhanced BioBERT (E-BioBERT), and widely-used bi-directional LSTM [29] are used as the encoder to yield contextualized word representations from input sentences. Then a simple convolution layer is used to build and refine the representation of the word-pair grid for later word-word relation classification. Afterward, a multi-layer perceptron is leveraged for reasoning the relations between all word pairs.

**Fig. 3:**
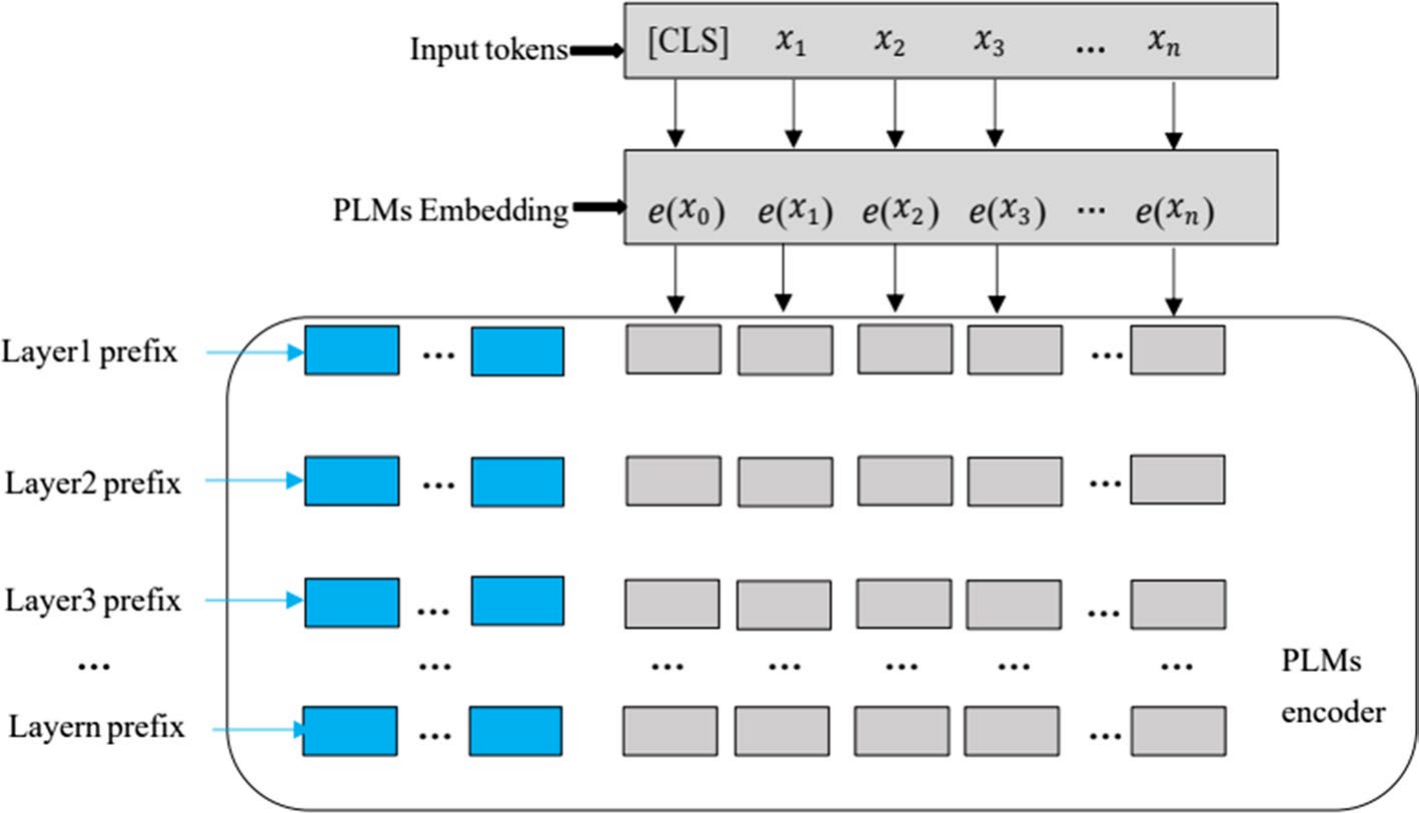
Prefix construction. We use the BioBERT as the PLM

#### Encoder layer

Answer engineering has a strong impact on the performance of prompt learning. As for entity class prediction in BioNER, adding additional label-specific parameters representing different entity types hinders the applicability of prompt learning [41, 72]. As shown in Fig. 3, we use the prefix tuning to tune the attention weights of BioBERT. This approach eliminates the need for a verbalizer and becomes a fully generative model that outputs a token-level class at each token position. Prompts in different layers are added as prefix tokens in the input sequence and are independent from other layers (rather than computed by previous transformer layers). Inspired by Chen et al. and Li et al. [41, 71], we add a set of trainable embedding matrices $\{\phi _{1},\phi _{2},\ldots ,\phi _{l}\}$ to each layer of BioBERT, where *l* is the layer number of BioBERT and $\phi _{\theta } \in {\mathbb {R}}^{P \times d }$ (*P* is the length of the prompt and *d* represents the dimension of the hidden layer of the encoder). The prefix of each layer participates in the calculation of self-attention. That is, unlike methods that place templates in the original input sequence, we incorporate continuous prompts into the self-attention layer and utilize these prefixes to guide attention allocation, which is sufficiently flexible and lightweight. Specifically, we inherit the structure of the Transformer, as a specific component, we introduce the prefix-guided attention layer over the original layer *queries*, *keys* and *values* (${\textbf {Q}}$, ${\textbf {K}}$, and ${\textbf {V}}$) to achieve more guided attention effect. As we all know, Transformer use stacked self-attentions to encode contextual information for input tokens [48]. The calculation of self-attention depends on the following components of **Q**, **K** and **V**, which are projected from the hidden vectors of the previous layer. Then the attention output **A** of one head is computed as follows:

1 $$\begin{aligned} {\textbf {A}} = softmax\left( \frac{{\textbf {Q}}{} {\textbf {K}}^{T}}{\root \of {{d}}}\right) {\textbf {V}} \end{aligned}$$

where d is the dimension of *keys*. Within the standard self-attention layer, global attention mechanism is employed that each token provides information to other tokens in the input sentence. A key feature of the Transformer architecture is the so-called multi-head attention mechanism, which allows the model to focus simultaneously on different parts of the input. Furthermore, Transformer rely on multi-head self-attention to capture dependencies between tokens. Given a hidden state **H** (input of initialized BioBERT), multi-head self-attention first projects it linearly into *queries* ${{\textbf {Q}}_{h}}$, *keys* ${{\textbf {K}}_{h}}$, and *values* ${{\textbf {V}}_{h}}$ using parameter matrices ${\textbf {W}}_{h}^{Q}$, ${\textbf {W}}_{h}^{K}$ and ${\textbf {W}}_{h}^{V}$ respectively. The formulation is as follows:

2 $$\begin{aligned} {\textbf {Q}}_{h}, {\textbf {K}}_{h}, {\textbf {V}}_{h} = {\textbf {HW}}_{h}^{Q}, {\textbf {HW}}_{h}^{K}, {\textbf {HW}}_{h}^{V} \end{aligned}$$

Then, we introduce the attention mechanism after the prefix to redefine the self-attention mechanism of ${\textbf {A}}_{h}$ as follows:

3 $$\begin{aligned} {\textbf {A}}_{h} =softmax\left( \frac{{\textbf {Q}}_{h}[{\textbf {K}}_{h};\phi _{k}^{h}]^{T}}{\root \of {{d}}}\right) [{\textbf {V}}_{h};\phi _{v}^{h}] \end{aligned}$$

where the self-attention distribution (attention weight) ${\textbf {A}}_{h}$ is computed via scaled dot-product of ${\textbf {Q}}_{h}$ and ${\textbf {K}}_{h}$ by Eq. 3. These weights are assigned to the corresponding *value* vectors ${\textbf {V}}_{h}$ to obtain output states ${\textbf {O}}_{h}$:

4 $$\begin{aligned} {\textbf {O}}_{h} = {\textbf {A}}_{h}{} {\textbf {V}}_{h} \end{aligned}$$.

**Fig. 4:**
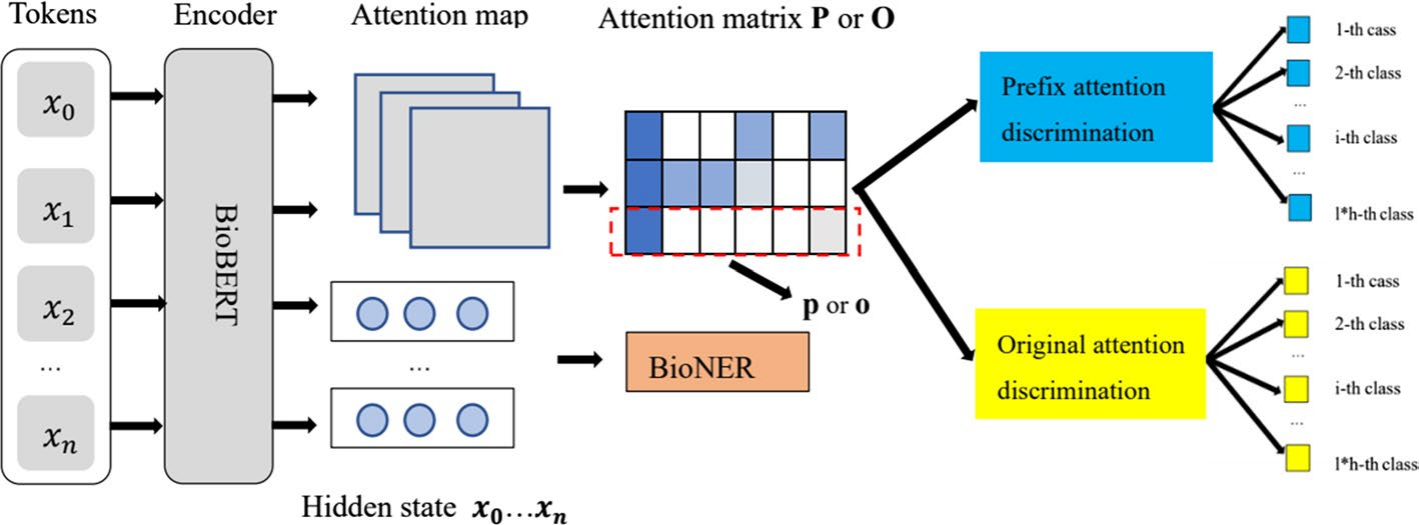
Our proposed attention. We fused the calculated loss of **p** and **o**

Finally, the output states ${\textbf {O}}_{h}$ of all heads are concatenated to produce the final states. To allow the different attention heads to interact with each other, Transformer applies a non-linear feed-forward network over the multi-head attention’s output at each Transformer layer. However, even with prefix-guided attention, for the BioNER task, we still find that our attention mechanism has redundant attention patterns and insufficient attention to entities. In view of the shortage of Transformer, we propose the PAMDFGA. Inspired by the instance discrimination learning proposed by Wu et al. [73], take BioBERT’s twelve layers and twelve heads as an example, we treat each head in BioBERT as an instance and match different heads at different layers to maximize the difference between different heads. This will make our model get more information from the input text from different aspects and perspectives. We want to learn a good feature representation for each instance (head), which requires the semantic information learned between different heads is as different as possible. Instance discrimination learning can implicitly group similar instances together in the representation space without any explicit learning force directs to do so [74]. Our designed attention discrimination model is shown in Fig. 4. The specific construction process of our designed attention PAMDFGA is as follows: we first obtain attention weights from different heads and layers of prefix-guided BioBERT. Our proposed attention mechanism is based on the whole attention map. This is expressed as follows:

5 $$\begin{aligned} \{{\textbf {A}}_{1}, {\textbf {A}}_{2}\ldots {\textbf {A}}_{i}, {\textbf {A}}_{i+1}\ldots {\textbf {A}}_{l*h}\}= BioBERT(x_{i} | \theta _{BioBERT}) \end{aligned}$$

where $\{{\textbf {A}}_{1}, {\textbf {A}}_{2}\ldots {\textbf {A}}_{i}, {\textbf {A}}_{i+1}\ldots {\textbf {A}}_{l*h}\}$ is BioBERT’s multi-head attention map. *l* and *h* denote the layer number and head number in each layer, respectively. Each attention map ${\textbf {A}}_{i}$ $\in$ ${\mathbb {R}}^{L \times (L+P)}$, where *L* is the maximum sentence length in each batch and *P* is the length of random initial prefix. $x_{i}$ represent the input token and $\theta _{BioBERT}$ is the trainable parameters of the BioBERT model which is fine-tuned during model training. Then we stack twelve layers of attention map and perform an average pooling operation on the $\textbf{A}_{i}$ by summing up the attention values that all **o**riginal (**o**) input length tokens received and all original input tokens with prefix (${\textbf {p}}$) received. Then, the corresponding formula of transforming the attention map ${\textbf {A}}_{i}$ to the attention vector ${\textbf {o}}_{i}$ and ${\textbf {p}}_{i}$ via the following:

6 $$\begin{aligned} \begin{aligned} {\textbf {o}}_{i}&= \sum _{j}^{L}{{\textbf {A}}_{i,j}} \\ {\textbf {p}}_{i}&= \sum _{j}^{L+P}{{\textbf {A}}_{i,j}} \end{aligned} \end{aligned}$$

where *i* represents the *i*-th attention map and *j* is the column index of ${\textbf {A}}_{i}$ of the attention map. ${\textbf {o}}_{i}$ $\in$ ${\mathbb {R}}^{L}$ and ${\textbf {p}}_{i}$ $\in$ ${\mathbb {R}}^{L+P}$. Then, we rebuild the entire attention map as follows:

7 $$\begin{aligned} \begin{aligned} {\textbf {O}}&= {\textbf {o}}_{1} \oplus {\textbf {o}}_{2} \oplus \,\cdots , \oplus \ {\textbf {o}}_{i} ,\ldots , \oplus \ {\textbf {o}}_{l*h} \\ {\textbf {P}}&= {\textbf {p}}_{1} \oplus {\textbf {p}}_{2} \oplus \,\cdots , \oplus \ {\textbf {p}}_{i} ,\ldots , \oplus \ {\textbf {p}}_{l*h} \end{aligned} \end{aligned}$$

where $\oplus$ denotes the concatenate operation. $\textbf{O}$ $\in$ ${\mathbb {R}}^{(l*h) \times L}$ and ${\textbf {P}}$ $\in$ ${\mathbb {R}}^{(l*h) \times (L+P)}$ represents the attention matrix. Finally, we push the diversity of attention maps via the idea of instance discrimination [73]. We treat each attention head as a distinct class of its own and train final category results of each head are different, so that the characteristics of each head are different, which means that the information of each head is different. The probability of one attention map ${\textbf {o}}$ and ${\textbf {p}}$ being assigned into the *i*-th class can be computed as follows:

8 $$\begin{aligned} \begin{aligned} {\textbf {O}}(i|{\textbf {o}})&=\frac{{exp}({\textbf {o}}_{i}^{T}{} {\textbf {o}}/\tau ) }{\sum _{j=1}^{{l*n}}{exp}({\textbf {o}}_{j}^{T}{} {\textbf {o}}/\tau )} \\ {\textbf {P}}(i|{\textbf {p}})&=\frac{{exp}({\textbf {p}}_{i}^{T}{} {\textbf {p}}/\tau ) }{\sum _{j=1}^{{l*n}}{exp}({\textbf {p}}_{j}^{T}{} {\textbf {p}}/\tau )} \end{aligned} \end{aligned}$$

where ${\textbf {o}}_{j}^{T}{} {\textbf {o}}$ measures how well **o** matches the *i*-th class because ${\textbf {o}}_{j}$ is regarded as the weight of *j*-th class. $\tau$ is a temperature parameter that controls the concentration of the distribution [75], which is necessary for tuning the concentration of ${\textbf {o}}$ on our unit sphere and we enforce $||{\textbf {p}}||$ and $||{\textbf {o}}||$ to 1 via a L2-normalization layer [73]. The objective of the auxiliary task is to maximize the joint probability $\prod _{i=1}^{l*h}P_{\theta }(i|f_{\theta }({\textbf {p}}_{i})$ and $\prod _{i=1}^{l*h}P_{\theta }(i|f_{\theta }({\textbf {o}}_{i})$ or equivalently to minimize the negative log-likelihood over the training set [51], as

9 $$\begin{aligned} \begin{aligned} Loss_{p}&= -\sum _{i=1}^{l*n}log P(i|f_{\theta }{({\textbf {p}}_{i}})) \\&=-\sum _{i=1}^{l*n}log(\frac{{exp}({\textbf {p}}_{i}^{T}{} {\textbf {p}}/\tau ) }{\sum _{j=1}^{l*n}exp({\textbf {p}}_{j}^{T}{} {\textbf {p}}/\tau )})\\ Loss_{o}&= -\sum _{i=1}^{l*n}log P(i|f_{\theta }{({\textbf {o}}_{i}})) \\&=-\sum _{i=1}^{l*n}log(\frac{exp({\textbf {o}}_{i}^{T}{} {\textbf {o}}/\tau ) }{\sum _{j=1}^{l*n}exp({\textbf {o}}_{j}^{T}{} {\textbf {o}}/\tau )})\\ \end{aligned} \end{aligned}$$

As such, the training objective of our PAMDFGA is revised as:

10 $$Loss_{{PAMDFGA}} = (Loss_{p} + Loss_{o} )/2$$

where $Loss_{PAMDFGA}$ fuse the information of the prefix and the original attention weight. We use $Loss_{PAMDFGA}$ as the auxiliary loss of main task $Loss_{BioNER}$.

Combined with our attention guidance mechanism, at the beginning of Fig. 3, using BioBERT, we add a special symbol token, i.e. ’[CLS]’, in front of each input sample [76]. By concatenating with both position embeddings and segmentation embeddings, the token embeddings were fed into the E-BioBERT model to get the output representation ${\varvec{{e(x}}}_{i})$ $\in$ ${\mathbb {R}}^{d_{x}}$. Formally, given the input tokens, the label-specific encoder calculates:

11 $$\begin{aligned} \begin{aligned}{}[{\varvec{{e(x}}}_{0}),{\varvec{{e(x}}}_{1}),\ldots ,{\varvec{{e(x}}}_{n})] = E-B ioBERT ([x_{0},x_{1},\ldots ,x_{n}];\theta _{E-BioBERT}) \end{aligned} \end{aligned}$$

where $\theta _{E-BioBERT}$ is the trainable parameters of the E-BioBERT model, which is fine-tuned during training. ${x_0}$ is the special token ’[CLS]’ and $d_{x}$ = 768 is the dimensionality of the local representation. Besides, we use the bi-directional LSTM [29] to yield contextual word representation from input embedding. The contextualized sentence-level representation $[{\varvec{{e(x}}}_{0}),{\varvec{{e(x}}}_{1}),\ldots ,{\varvec{{e(x}}}_{n})]$ are used as the input embeddings of bi-directional LSTM layer, denoted as,

12 $$\begin{aligned} \begin{aligned}{}[{\varvec{{h}}}_{0},{\varvec{{h}}}_{1},\ldots ,{\varvec{{h}}}_{n}] = BiLSTM([{\varvec{{e(x}}}_{0}),{\varvec{{e(x}}}_{1}),\ldots ,{\varvec{{e(x}}}_{n})];\theta _{BiLSTM}) \end{aligned} \end{aligned}$$

where $\theta _{BiLSTM}$ is the corresponding trainable parameters of the BiLSTM model. ${\varvec{{h}}}_{i}$ $\in$ ${\mathbb {R}}^{d_{h}}$, where ${d_{h}}$ denotes the dimension of a word representation. $[{\varvec{{h}}}_{0},{\varvec{{h}}}_{1},\ldots ,{\varvec{{h}}}_{n}]$ are the hidden layer state sequence of BiLSTM [76].

#### Convolution layer

The second part of the model is the convolutional layer. Since CNNs are naturally suitable for 2-dimensional convolution on the grid, and also show the very prominence on handling relation determination jobs [39, 77]. We use a convolution module to capture grid information. Our convolution layer includes three modules, including a condition layer with normalization [39, 78] (CLN) for generating the representation of the word-pair grid, a hybrid sentence grid representation build-up to enrich the representation of the word-pair grid, and a single-layer dilated convolution for capturing the interactions between close and distant words. Specifically, we follow prior work [39, 78] and use a conditional layer with normalization for generating the representation of the word-pair grid. Then, we combine the enhanced word pair representations from CLN with randomly initialized distance and region embeddings to augment sentence representations. In the third part of the convolutional layer, we just use a simple and single-layer dilated convolutional neural network to capture the interaction information between different word pairs. The specific module information of the convolutional layer is as follows.

*Conditional layer normalization*

The idea of conditional layer normalization comes from the idea of popular conditional generative adversarial networks (GAN) in image field - conditional batch normalization (CBN). That means a conditional vector is introduced as external contextual information to generate the gain parameter and bias of the well known layer normalization [79] (LN) mechanism. In our BioNER framework, since we need to to predict the final relations between word pairs by generating grid representations of the word-pair grid, which can be regarded as a 3-dimensional matrix, ${\textbf {W}}$ $\in$ ${\mathbb {R}}^{(N \times N \times d_{h})}$, where ${\textbf {W}}_{ij}$ denotes the representation of the word pair $({x}_{i},{x}_{j})$ and N is the number of tokens in each batch. Because both NNW and THW relations are directional, the representation ${\textbf {W}}_{ij}$ of the word pair $({x}_{i},{x}_{j})$ can be considered as a combination of the representation ${\varvec{{h}}}_{i}$ of ${x}_{i}$ and ${\varvec{{h}}}_{j}$ of ${x}_{j}$, where the combination should imply that ${x}_{j}$ is conditioned on ${x}_{i}$. We adopt the CLN to calculate ${\textbf {W}}_{ij}$:

13 $$\begin{aligned} \begin{aligned} {\textbf {W}}_{ij}&= CLN({\varvec{{h}}}_{i},{\varvec{{h}}}_{j}) \\&= \gamma _{ij}\odot \left( \frac{{\varvec{{h}}}_{j}-\mu }{\sigma }\right) + \lambda _{ij} \end{aligned} \end{aligned}$$

where ${\varvec{{h}}}_{i}$ is the condition to generate the gain parameter $\gamma _{ij}={\textbf {W}}_{\alpha }{\varvec{{h}}}_{i}+{\textbf {b}}_{\alpha }$ and bias $\lambda _{ij}={\textbf {W}}_{\beta }{\varvec{{h}}}_{i}+{\textbf {b}}_{\beta }$ of layer normalization. $\mu$ and $\sigma$ are the mean and standard deviation across the elements of ${\varvec{{h}}}_{j}$, denoted as:

14 $$\begin{aligned} \begin{aligned} \mu&= \frac{1}{d_{h}}\sum _{k=1}^{d_{h}}h_{jk} \\ \sigma&= \sqrt{\frac{1}{d_{h}}\sum _{k=1}^{d_{h}}(h_{jk}-\mu )^{2}} \end{aligned} \end{aligned}$$

where $h_{jk}$ denotes the *k*-th dimension of ${\varvec{{h}}}_{j}$ [39].

*Hybrid sentence representation*

Building a grid representation of word pairs is a key step in our word pair classification. To further enhance sentence representations from E-BioBERT and conditional layer normalization, the distance embeddings (**D**) and region embeddings (**R**) are leveraged to better represent the positional information of word pairs in the grid. After we get the 3-dimensional vector ${\textbf {W}}$ $\in$ ${\mathbb {R}}^{N \times N \times d_{h}}$ encoded by the BiLSTM encoder and E-BioBERT, we concat the word embedding (${\textbf {W}}$), distance embedding (${\textbf {D}}$), and region embedding (${\textbf {R}}$) together. ${\textbf {D}}$ and ${\textbf {R}}$ are also 3-dimensional vectors, where ${\textbf {D}}$ $\in$ ${\mathbb {R}}^{N \times N \times d_{d}}$ and ${\textbf {R}}$ $\in$ ${\mathbb {R}}^{N \times N \times d_{r}}$. Finally, we concatenate these three vectors to enhance the region and distance information of the hybrid sentence representation of grid ${\textbf {G}}$ $\in$ ${\mathbb {R}}^{N \times N \times d_{g}}$. The overall process can be formulated as:

15 $$\begin{aligned} {\textbf {G}} = MLP([{\textbf {W}}\otimes {\textbf {R}}\otimes {\textbf {D}}]) \end{aligned}$$

where MLP is a multi-layer perception to reduce their dimensions and $\otimes$ represents concatenation operations.

*Convolutional neural network*

Convolutional neural network is generally used in the field of computer vision for tasks such as image classification and detection. The core idea of CNN is to capture local features. For text, local features are sliding windows composed of several words, similar to N-gram. The advantage of CNN is that it can automatically combine and filter N-gram features to obtain semantic information at different levels of abstraction. This is beneficial for enriching the semantic information of ${\textbf {G}}$. We use a single layer dilated convolutional neural network (*SDConv*) to capture the interactions between word pairs, denoted as:

16 $$\begin{aligned} {\textbf {S}} = \sigma (SDConv({\textbf {G}})) \end{aligned}$$

where **S** $\in$ ${\mathbb {R}}^{N \times N \times d_{g}}$ and $\sigma$ is the GELU activation function [80].

#### Classifier

Our model mainly predicts the relationship of word pairs, that is, the probability that a directed graph edge belongs to a category. The vector ${\textbf {S}}$ from *SDConv* represents the grid information of word pairs, and we use the MLP to calculate two separate relations scores of word pair ($x_{i}$, $x_{j}$) and use the Softmax function to calculate the final relation probabilities, using ${\textbf {S}}_{ij}$,

17 $$\begin{aligned} {\textbf {y}}_{ij} = softmax (MLP({\textbf {S}}_{ij})) \end{aligned}$$

where $MLP({\textbf {S}}_{ij})$ $\in$ ${\mathbb {R}}^{|\mathfrak {R}|}$ is the scores of the relations pre-defined in $\mathfrak {R}$. Finally, for the $Loss_{BioNER}$, our BioNER training target is to minimize the negative log-likelihood losses with regards to the corresponding gold labels, formalized as:

18 $$\begin{aligned} \begin{aligned} Loss_{BioNER} = -\frac{1}{N^2}\sum _{i=1}^{N}\sum _{j=1}^N\sum _{r=1}^{|\mathfrak {R}|}\hat{{\textbf {y}}}^{r}_{ij}log{\textbf {y}}^{r}_{ij} \end{aligned} \end{aligned}$$

where N is the number of words in the sentence, $\hat{{\textbf {y}}}_{ij}$ is the binary vector that denotes the gold relation labels for the word pair ($x_{i}$,$y_{j}$), and ${\textbf {y}}_{ij}$ are the predicted probability vector. *r* indicates the *r*-th relation of the pre-defined relation set $\mathfrak {R}$. As such, our total training target is to minimize the loss of BioNER and the loss of PAMDFGA, formalized as:

19 $$Loss_{{Total}} = Loss_{{BioNER}} + \alpha Loss_{{PAMDFGA}}$$

where $Loss_{PAMDFGA}$ is defined in Eq. 10 and $\alpha Loss_{PAMDFGA}$ can be seen as a regularization loss, which are regulated using $\alpha$, and this term works like L2 term which does not introduce any new parameters and only influence the fine-tuning of the standard model parameters [51].

#### Decoding

The five BioNER datasets used in our framework are all flat NER. For the word-pair relationship scores predicted by the framework, we decode our predictions as a directional graph. The decoding object is to find certain paths from one word to another word in the graph using NNW relations. THW is used to determine the boundaries and type of entities, especially for sentences without entities, our THW is empty, and we do not judge which category it belongs to. Specifically, the relationships $\mathfrak {R}$ of all the word pairs serve as the inputs. The decoding object is to find all the entity word index sequences with their corresponding categories. First, since our dataset has no nested examples, in the lower triangle part of Fig. 1, we can decode them out just using THW-$\star$. For multiple consecutive entities, we construct a graph to, in which nodes are words and edges are NNW relations. Then we use the deep first search algorithm to find all the paths from the head word to the tail word, which are the word index sequences of corresponding entities [39].

## Results

### Datasets and metrics

We evaluate our model on five public and available datasets containing various biomedical entities: BC4CHEMD [81], BC5CDR [82] (including two sub-datasets, BC5CDR-Disease and BC5CDR-Chem), NCBI-Disease [83], BC2GM [84] and, all of which are pre-processed and provided by previos SoTA work. Table 1 summarizes these datasets. Among them, BC4CHEMD has the most sentences and entities and NCBI-Disease has the least datasets. As the same with previous work [18, 22, 23, 25, 26], we merged the train and development sets, made the same data split, and evaluated our model on the test set for a fair comparison. We follow prior SoTA works [25, 26], and adopt standard entity-level F1-score as evaluation metrics to measure the performance of the trained model. Specifically, a predicted entity is counted as true-positive if its token sequence and type match those of a gold entity. The corresponding metrics are Precision ($\mathrm P$), Recall ($\mathrm R$), and F1-score ($\mathrm F1$), where $\mathrm F1 = 2 \times \mathrm P \times \mathrm R/(\mathrm P + \mathrm R)$.

**Table 1:** Datasets description

Datasets	Entity type	Annotations	Sentences
BC4CHEMD	Chemical/Drug	84,130	87,685
BC2GM	Protein/Gene	20,131	24,583
BC5CDR-Disease	Disease	12,852	13,938
BC5CDR-Chem	Chemical/Drug	15,935	13,938
NCBI-Disease	Disease	6881	7287

### Settings

The BioBERTv1.1 (+PubMed, Cased) [20] model was used, containing 12 layers of Transformers with a hidden size of 768. The dimensionality of the hidden state $d_{h}$ in BiLSTM is 512, the channel size of the convolutional layer $d_{g}$ is set to 128 and the size of distance embedding and region embedding is initialized to 20. All datasets are trained with the batch size of 8 except BC4CHEMD, which has a batch size of 4. We use the AdamW optimizer [85] with a learning rate 1e-3 for all datasets. We select the sentence length of the largest sample in each batch for training. A linear learning rate decay schedule with warm-up over 0.1, and a weight decay of 0.1 applied to every epochs of the training [26]. The $\alpha$ in Eq. 19 are selected from the set {0.1, 0.01, 0.001, 0.0001} according to grid search. The temperature parameter is set to 2.0 [51]. On all the datasets, each experiment is repeated five times. We report the maximum F1-score (referred to “Max”), average F1-score (referred to “Mean”), and standard deviation (referred to “Std”). Table 2 demonstrates our work. The proposed attention guiding mechanism acts on all attention heads of BioBERT. The best results in all our datasets are obtained based on integrating PAMDFGA into the last four layers of BioBERT. The best training procedure contains 6 epochs for BC4CHEMD, 10 epochs for BC2GM, 41 epochs for NCBI-Disease, 34 epochs for BC5CDR-Disease, and 47 epochs for BC5CDR-Chem. All our ablation study and case study are performed under the same parameters and epochs. Because the model training is not complicated, we do not freeze the parameters of BioBERT. Finally, all models are trained on NVIDIA RTX 3090.

**Table 2:** Experimental results over five runs

Datasets	Mean ± Std	Max
BC4CHEMD	92.55 ± (0.00)	**92.55**
BC2GM	85.35 ± (0.06)	**85.45**
BC5CDR-Disease	87.51 ± (0.02)	**87.53**
BC5CDR-Chem	94.10 ± (0.06)	**94.16**
NCBI-Disease	90.40 ± (0.08)	**90.55**

“Max” denotes the maximum F1-score, “Mean” denotes the average F1-score, and “Std” denotes the standard deviation. The best scores are shown in bold

### Performance and comparisons

We compare our model with a wide range of methods. These methods are based on sequence tagging. To be specific, we compare our model with the approaches based on neural network [16–18], approaches based on pre-trained language models, such as BERT [19] and BioBERT [20], approaches based on external knowledge [10, 21] and the approaches based on mulit-task learning [22–26]. As can be seen from Table 3, multi-task learning in solving BioNER tasks is becoming more and more popular, among them, Chai et al. [25] achieved SoTA performance on two datasets BC4CHEMD and BC5CDR-Chem by training a model on 14 datasets, which realizes the multi-level information fusion between the underlying entity features and the upper data features. Both multi-task learning and fine-tuning are applied to their model. Tong et al. [26] design multiple auxiliary classification losses by incorporating multi-granularity information in the datasets to achieve the best performance in the BC4CHEMD, BC5CDR-Chem, and BC5CDR-Disease datasets. They all get the best performance without utilizing additional resources. It is worth noting that Tian et al [10] injects a lot of external syntactic knowledge (i.e., POS labels, syntactic constituents, and dependency relations) into BioBERT in the form of a key-value pair that works best on the BC2GM, BC5CDR-Chem and NCBI-Disease datasets. Although additional knowledge and multi-task learning can alleviate the problem of insufficient data, the additional knowledge usually contains a lot of noise, and it is difficult for us to control how much additional information should be selected. The training process of multi-task learning is too complicated, so it is difficult for us to design a general multi-task framework for many BioNER datasets. Therefore, the current methods can only be effective on some specific datasets. However, surprisingly, we achieve the best performance on all five datasets by using a novel word-pair relation classification schema and the proposed PAMDFGA. As indicated in Table 3, compared with the models without additional knowledge, the improvement effect of our model is more obvious. First, we can see that our model outperforms existing methods, regardless of whether they introduce external knowledge, which further confirms the validity of our innovation in enhancing BioNER feature extraction. Second, although some models utilize higher-level features, such as Tian et al [10]. leverages POS tags, syntactic constituents, and dependencies rules, and Tong et al. [26] employs multi-task learning to train the model, our model can achieve better results with a simple attention guiding. This means our proposed model can better mine semantic information and solve entity sparse problems in all datasets, especially when mining datasets of disease entities (NCBI-Disease and BC5CDR-Disease) and gene entities (BC2GM). We can conclude that the features extracted from the PAMDFGA module are effective in assisting biomedical text representation, and even show more potential than special designs in the biomedical field, with the whole attention map guidance being pushed.

**Table 3:** Model performance comparison on the five benchmark datasets

Model	BC4CHEMD			BC2GM			BC5CDR-Disease			BC5CDR-Chem			NCBI-Disease		
	$\textbf{P}$	$\textbf{R}$	$\textbf{F1}$	$\textbf{P}$	$\textbf{R}$	$\textbf{F1}$	$\textbf{P}$	$\textbf{R}$	$\textbf{F1}$	$\textbf{P}$	$\textbf{R}$	$\textbf{F1}$	$\textbf{P}$	$\textbf{R}$	$\textbf{F1}$
Habibi et al. [16]	–	–	–	81.57	79.48	80.51	–	–	–	87.60	86.25	86.92	86.11	85.49	85.80
Luo et al. [17]	–	–	–	–	–	–	89.61	83.09	86.23	–	–	–	90.72	74.89	82.05
Sachan et al. [18]	–	–	–	81.81	81.57	81.69	–	–	–	–	–	–	86.41	88.31	87.34
Devlin et al. [19]	–	–	–	81.17	82.42	81.79	–	–	–	90.94	91.38	91.16	84.12	87.19	85.63
Lee et al. [20]	92.80	91.92	92.36	84.32	85.12	84.72	86.47	89.84	87.15	93.68	93.26	93.47	88.22	91.25	89.71
Yu et al. [21]	–	–	–	–	–	84.52	–	–	85.62	–	–	93.33	–	–	87.82
Tian et al. [10]	–	–	–	–	–	84.92	–	–	–	–	–	94.00	–	–	90.08
Wang et al. [22]	90.78	87.53	89.37	82.10	79.42	80.74	–	–	–	–	–	–	85.86	86.42	86.14
Yoon et al. [23]	–	–	–	80.49	78.99	79.93	–	–	–	94.26	92.38	93.31	85.48	87.27	86.36
Khan et al. [24]	–	–	–	82.10	84.04	83.01	–	–	–	88.46	90.52	89.48	86.73	89.70	88.19
Chai et al. [25]	–	–	92.42*	–	–	82.92	–	–	87.28*	–	–	93.83	–	–	89.25
Tong et al. [26]	–	–	–	84.42	85.14	84.78*	–	–	–	93.29	94.69	93.98*	88.90	90.04	89.91*
PAMDFGA	91.74	93.37	**92.55**	85.43	85.47	**85.45**	87.11	87.95	**87.53**	93.66	94.67	**94.16**	89.76	91.35	**90.55**

We separate the comparison methods of different categories with horizontal lines

* indicates the best result without using additional knowledge. _ denotes the best result integrating external knowledge

P is the Precision, R is the Recall, and F1 means the F1-score

Data are expressed in percentage signs ($\%$). Bold F1-score represent our results

**Table 4:** Ablation study

Model	Dataset														
	BC4CHEMD			BC2GM			BC5CDR-Disease			BC5CDR-Chem			NCBI-Disease		
	P	R	F1	P	R	F1	P	R	F1	P	R	F1	P	R	F1
Baseline	91.07	93.80	92.42	86.27	83.57	84.90	85.87	88.45	87.14	94.51	93.70	93.61	88.81	90.10	89.45
PAMDFGA w/o $Loss_{o}$	92.56	92.67	**92.62**	85.13	84.79	84.96	86.47	87.97	87.22	93.31	94.30	93.80	88.29	91.15	89.70
PAMDFGA w/o $Loss_{p}$	92.19	92.13	92.16	85.63	84.89	85.26	86.63	88.31	87.46	93.64	94.56	94.10	89.02	91.25	90.12
PAMDFGA	91.74	93.37	92.55	85.43	85.47	**85.45**	87.11	87.95	**87.53**	93.66	94.67	**94.16**	89.76	91.35	**90.55**

Bold F1 represents the best result

Baseline means not using PAMDFGA and its components

## Discussion

### Ablation study

Since our proposed attention mechanism PAMDFGA performs an average fusion of the attention weights in the two dimensions of prefix and original text length. In order to analyze the impact of different components of our proposed attention mechanism on different datasets. As shown in Table 4, we conducted ablation experiments under the same experimental parameters, and the pre-trained model we used was BioBERT. In this table, baseline refers to our model not using any attention mechanism. PAMDFGA w/o $Loss_{p}$ refers to not using $Loss_{p}$ but retaining $Loss_{o}$, PAMDFGA w/o $Loss_{o}$ refers to not using $Loss_{o}$ but retaining $Loss_{p}$, and PAMDFGA refers to the final attention proposed. It can be seen from the results in Table 4 that different components have certain guiding roles for BioBERT. Among them, PAMDFGA is better than using a single component alone in BG2GM, BC5CDR-Disease, BC5CDR-Chem and NCBI-Disease datasets. In view of the dataset BC4CHEMD, our proposed guidance does not perform as well as using the original attention weights. The reasons are two-fold: (1) this dataset, BC4CHEMD, are large in scale and the sentences in training set without entities are too long and too large. (2) After random initialization of BioBERT, the prefix distribution is mapped to the high dimensional space, resulting in the entity distribution is too sparse. So the guiding effect of these words is not good. Besides, we can find that the impact without $Loss_{o}$ is bigger than without $Loss_{p}$. But for the fused attention mechanism, $Loss_{p}$ plays a further guiding role. However, compared with the baseline, our proposed three-way guided attention mechanism has significantly improved the entity recognition of the BioBERT because this attention mechanism can better utilize the grid information of word pair relationships. Removing any of them will result in performance degradation for almost all metrics. The results of ablation experiments demonstrate that our proposed PAMDFGA can bring more valuable information to BioBERT, as PAMDFGA can push each head to focus on different locations of the input to capture diversity information.

### Effect of different prefix length for PAMDFGA

**Fig. 5:**
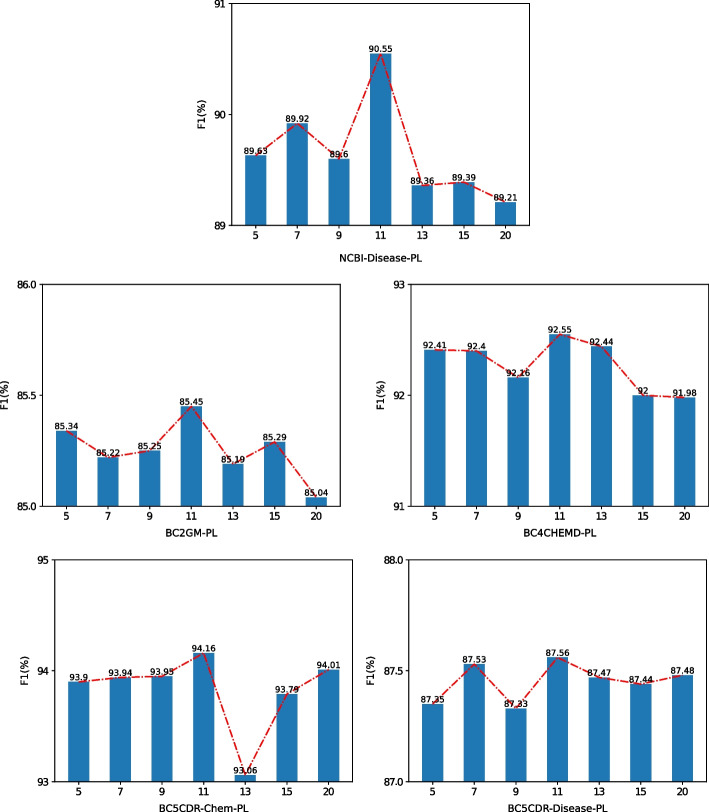
Performance of different prefix length. PL denotes prefix length

Prefix length (*P* in Eq. 7) is a influential hyper-parameter for PAMDFGA because the length of the prefix will participate in the calculation of self-attention, which will affect the effect of the words that PAMDFGA pays attention to. As shown in Fig. 5, we experimentally demonstrate that the length of the prefix is the best within 20. The prefix length are selected from the set {5, 7, 9, 11, 13, 15, 20} according to grid search in our experiments. Figure 5 illustrates that prefix length shows a similar distribution across the datasets, with a prefix length of 11 performing best on all datasets. This may be related to the sentence length in BioNER. As for this, the prefix length of 11 was chosen for both our ablation experiment and the best experimental results to guide our model.

### Effect of PAMDFGA on different layers of BioBERT

**Fig. 6:**
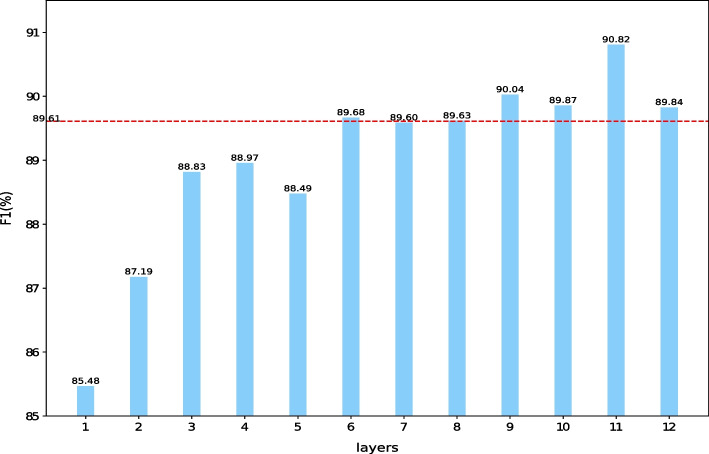
Performance of each BioBERT layer with PAMDFGA. The red dashed line indicates that we replicated the results of Lee et al. [20] on the NCBI-Disease dataset

To demonstrate that our proposed PAMDFGA can be better integrated into each layer of PLMs, we studied the effect of PAMDFGA on each layer of BioBERT. We conducted experiments on the NCBI-Disease test set. As shown in Fig. 6, for NCBI-Disease dataset, most layers in BioBERT can benefit from the proposed PAMDFGA, and the improvement effect is more obvious in the last four to five layers. Among them, the F1-score of the eleventh layer increased from 89.87 to 90.82%. The F1-score of the last layes has decreased relative to the eleventh layer, which is understandable because PAMDFGA encourages pushing information of different heads of BioBERT. That is, our attention mechanism plays a better attention effect in other layers. It can also be argued that PAMDFGA makes the attention information of different heads more diverse compared to the patterns that traditional pre-trained models pay attention to. That’s because traditional pre-trained models have incremental F1 in the last few layers. In all our final experiments, we integrate PAMDFGA into the last four layers of BioBERT. For comparison, the baseline system also uses the outputs of the last four layers of BioBERT.

### Computational cost analysis

Our proposed attention machine can work in the fine-tuning phase without modifying the self-attention formula, which means we does not need to re-train the PLMs. Our entire model maintains a time complexity identical to the Transformer, which is ${\mathcal {O}} (N^{2})$. *N* is the length of the input sentence. Thus, PAMDFGA also has merits in terms of time cost. Nevertheless, the calculation process of it will take more time than directly fine-tuning the pre-trained model. Table 5 shows the per-epoch training time of entire fine-tuned model on five datasets. As can be seen from Table 5, the increased time cost is minor by adding PAMDFGA. Specifically, the external time cost by PAMDFGA per-epoch training is about 2.21 s, 1.11 s, 3.35 s and 1.68 s on BC2GM, BC5CDR-Disease, BC5CDR-Chem and NCBI-Disease datasets, respectively. We can see that our attention mechanism hardly adds to the computational cost of training because we don’t introduce additional parameters. In term of this, the advantage of PAMDFGA is even more significant. For the dataset BC4CHEMD, the time of training time increases 24.74 s. We consider the time cost acceptable, since this dataset is inherently large and PAMDFGA improves the recognition of chemical disease terms.

**Table 5:** Per-epoch training time (in seconds) with or without PAMDFGA

Datasets	Baseline	Ours	Time increased
BC4CHEMD	1656.22	1680.96	24.74
BC2GM	250.49	252.70	2.21
BC5CDR-disease	146.89	148.00	1.11
BC5CDR-chem	146.20	149.55	3.35
NCBI-disease	67.38	69.06	1.68

### Case study

To show and prove the validity of our proposed attention mechanism, we plot the full attention heatmaps on NCBI-Disease dataset to verify the reason why the 11-th layer works well in Fig. 6. And we perform qualitative analysis on BC5CDR-Chem dataset with their real labels and predicted labels from the method based on sequence labelling using BioBERT and our model.

#### Attention visualization

**Fig. 7:**
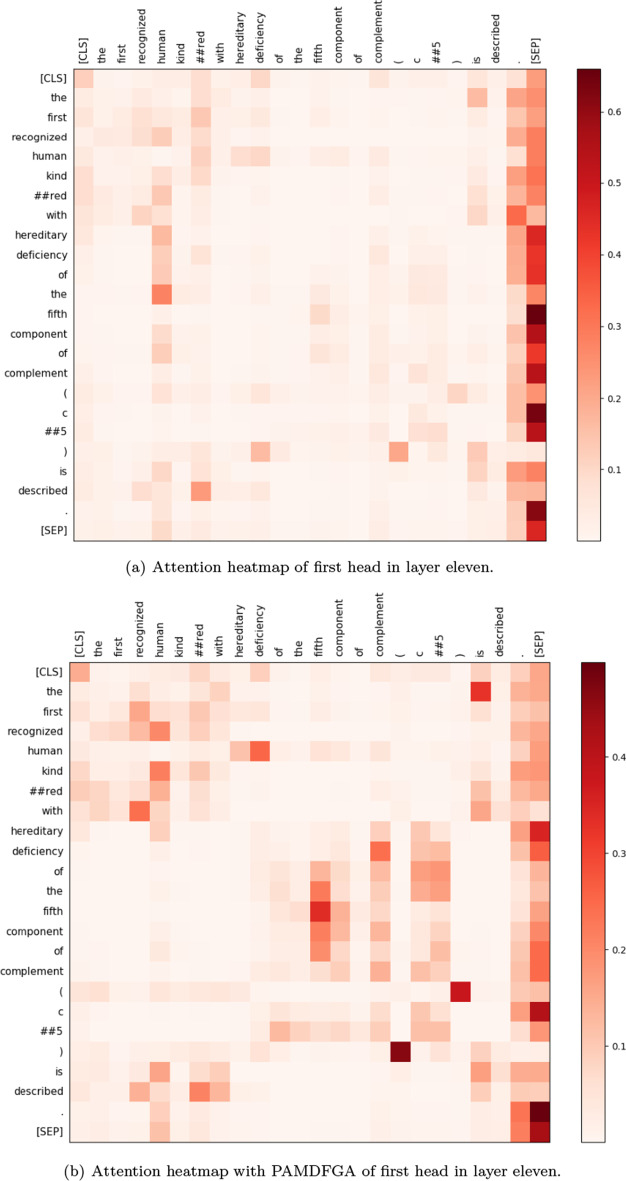
Visualization of attention scores over the first head in layer 11. This case is selected from the NCBI-Disease test set. Darker colors correspond to greater attention scores

**Fig. 8:**
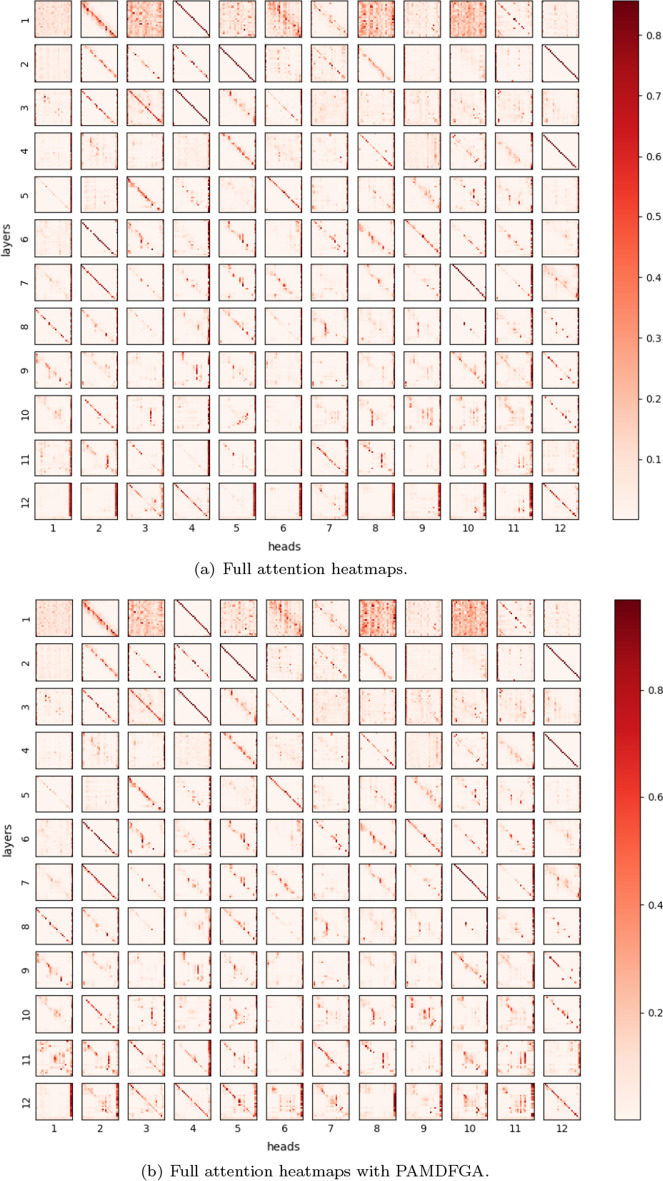
Visualization of attention scores over all heads and all layers. This case is selected from the NCBI-Disease test set. The ordinate indicates that BioBERT has 12 layers, and the ordinate indicates that each layer contains 12 heads. Darker colors correspond to greater performance

To show the validity of the PAMDFGA in Fig. 6 and prove our attention can pay attention to more positions, we present examples of full self-attention maps of a set of fine-tuned models with/without the PAMDFGA to provide a better illustration of different head pattern in Figs. 7 and 8. The selected token sequence from NCBI-Disease after the WordPiece tokenizer is “[’[CLS]’, ’the’, ’first’, ’recognized’, ’human’, ’kind’, ’##red’, ’with’, ’hereditary’, ’deficiency’, ’of’, ’the’, ’fifth’, ’component’, ’of’, ’complement’, ’(’, ’c’, ’##5’, ’)’, ’is’, ’described’, ’.’, ’[SEP]’]”. Specifically, we first take the attention weight change of the first head of BioBERT layer 11 as an example, as shown in Fig. 7, we can see that there are many informative tokens overlooked by the self-attention without PAMDFGA (Fig. 7a) but captured by our method (Fig. 7b). Looking further at the full attention map, as shown in Figure  8, we can find that the repeated attention patterns like diagonal pattern [47] of different heads in different layers of BioBERT after using PAMDFGA are significantly reduced, and different heads in the last four layers of BioBERT, the words that the attention head pays attention to are more diverse. In this way, the probability of the entity being noticed will naturally increase. We can conclude our attention mechanism pushes the diversity of the entire attention map. This is also explain why the F1-score of layer 11 in Fig. 6 is better because layer 11 pays more attention to the words near the entity words. For example, more attention is paid to the token ’hereditary’ and ’deficiency’. In fact, these tokens constitute an important biomedical entity, which should be paid more attention. Most other heads have a similar effect. The comparison of the heatmaps between the eleventh and twelfth layers in Fig. 8b also demonstrates why the F1-score of the eleventh layer is higher.

#### Qualitative analysis

**Fig. 9:**
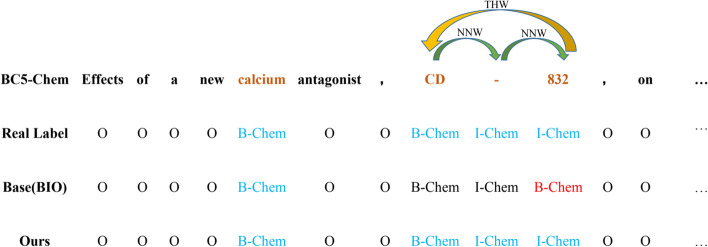
Examples of two predicted labels from sequence tagging (Base(BIO)) and our method (Ours). This case is selected from the BC5CDR-Chem test set. Orange indicates the corresponding entities. The blue word “B-Chem” represents the real label. Black “B-Chem” and red “B-Chem” represent labels that are correctly predicted and incorrectly predicted by the model, respectively. The green and yellow arrows represent the labels predicted by our method

**Fig. 10:**
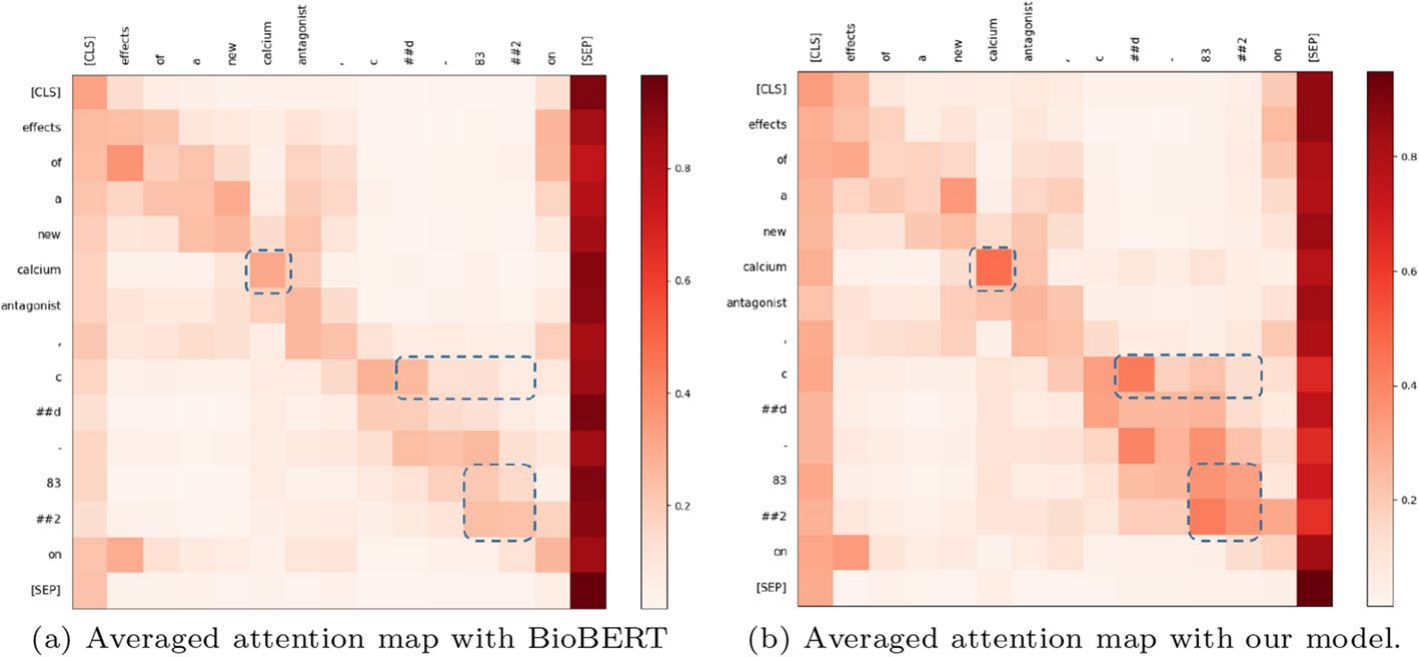
Visualization of attention scores averaged over all heads and all layers. This case is selected from the BC5CDR-Chem test set. The blue rectangle indicates higher scores on the right side but lower scores on the left side. Darker colors correspond to greater performance

We randomly sampled one sentence from the BC5CDR-Chem test set and compare the sequence tagging method using BioBERT with the word pair model with PAMDFGA. Figure 9 shows that our model has certain advantages over BioBERT in terms of learning entity information and alleviate the problem of label inconsistency. For example, BioBERT model based on sequence tagging usually only recognizes entity composed of a single word such as “calcium”, and the entity composed of multiple words such as “CD-832” tends to be identified incorrectly, causing the base model regards these words as two different entities. As we all know, “CD-832” is an important and complete chemical entity. But surprisingly, since word-pair relationship classification can better capture the relationship between adjacent entities, our model will recognize that “CD” and “-” and “-” and “832” are the relation of NNW. “832” and “CD” are the relation of THW. Our model thus decodes the identified relationship into a complete entity. To further verify how much our model pays attention to entities, we draw the attention heatmaps of the model from the average attention perspective in Figure  10. We mainly focus on the interactions of tokens, except for ’[CLS]’ and ’[SEP]’. The selected token sequence after the WordPiece tokenizer is “[’[CLS]’, ’Effects’, ’of’, ’a’, ’new’, ’calcium’, ’antagonist, ’c’, ’##D’, ’83’, ’##2’, ’[SEP]’]”. Then the attention scores are averaged over all heads and layers. This visualization validates the effectiveness of proposed attention compared with the traditional self-attention pattern. As shown in Fig. 10, we can see that there are many informative tokens overlooked by the Transformer-based method (Fig. 10a) but captured by our method (Fig. 10b). For instance, the PAMDFGA allows the tokens “CD” to strongly attend to the token “-” and “832”, but these tokens are paid less attention in the Transformer-based attention. In addition, our model also strengthens the attention between sub-words such as ’83’, ’##2’. These explain why our model can better capture the semantic information of neighbor words.

## Conclusion

In this work, we addresses the BioNER problem with a new prediction pattern for the first time. Experiments show that this prediction mode has better entity recognition ability and entity words modeling ability than sequence labeling. Empirically, to further improve the performance to recognize biomedical entities, we design a novel and efficient prefix and attention map discrimination fusion guided attention mechanism by changing the attention distribution to enhance BioBERT. And our method outperforms the four existing mainstream methods. This work also points to a promising direction and provides a new research angle for BioNER. As to future work, we plan to explore the effectiveness of PAMDFGA in different PLMs and different biomedical tasks, and explore how to incorporate more domain-specific knowledge to guide self-attention learning in other domains such as some biomedical low resource domains.

## Data Availability

The dataset is available on https://github.com/cambridgeltl/MTL-Bioinformatics-2016. Our code is released at https://github.com/Guan-cloud/PAMDFGA.

## Abbreviations

DL: Deep learning
BioNER: Biomedical named entity recognition
NER: Named entity recognition
PAMDFGA: A prefix and attention map discrimination fusion guided attention
CLN: Conditional layer normalization
CRF: Conditional random field
NLP: Natural language processing
NLI: Natural language inference
